# Mir-206 Regulates Pulmonary Artery Smooth Muscle Cell Proliferation and Differentiation

**DOI:** 10.1371/journal.pone.0046808

**Published:** 2012-10-10

**Authors:** Samuel Jalali, Gurukumar K. Ramanathan, Prasanna Tamarapu Parthasarathy, Salman Aljubran, Lakshmi Galam, Asfiya Yunus, Sara Garcia, Ruan R. Cox, Richard F. Lockey, Narasaiah Kolliputi

**Affiliations:** Division of Allergy and Immunology, Department of Internal Medicine, Morsani College of Medicine, University of South Florida, Florida, United States of America; Goethe University, Germany

## Abstract

Pulmonary Arterial Hypertension (PAH) is a progressive devastating disease characterized by excessive proliferation of the Pulmonary Arterial Smooth Muscle Cells (PASMCs). Studies suggest that PAH and cancers share an apoptosis-resistant state featuring excessive cell proliferation. MicroRNA-206 (miR-206) is known to regulate proliferation and is implicated in various types of cancers. However, the role of miR-206 in PAH has not been studied. In this study, it is hypothesized that miR-206 could play a role in the proliferation of PASMCs. In the present study, the expression patterns of miR-206 were investigated in normal and hypertensive mouse PASMCs. The effects of miR-206 in modulating cell proliferation, apoptosis and smooth muscle cell markers in human pulmonary artery smooth muscle cells (hPASMCs) were investigated in *vitro.* miR-206 expression in mouse PASMCs was correlated with an increase in right ventricular systolic pressure. Reduction of miR-206 levels in hPASMCs causes increased proliferation and reduced apoptosis and these effects were reversed by the overexpression of miR-206. miR-206 over expression also increased the levels of smooth muscle cell differentiation markers α-smooth muscle actin and calponin implicating its importance in the differentiation of SMCs. miR-206 overexpression down regulated Notch-3 expression, which is key a factor in PAH development. These results suggest that miR-206 is a potential regulator of proliferation, apoptosis and differentiation of PASMCs, and that it could be used as a novel treatment strategy in PAH.

## Introduction

Pulmonary arterial hypertension (PAH) is a disease characterized by a progressive increase in pulmonary vascular resistance, causing an increase in pulmonary artery blood pressure [1] leading to right heart failure and ultimately death. Both pulmonary artery smooth muscle cells (PASMCs) and pulmonary artery endothelial cells (PAECs) are affected by this disease. It is characterized by uncontrolled cell proliferation and increased resistance of PASMCs to apoptosis [1], [2]. Under PAH conditions, PAECs become dysfunctional through reduced production of nitric oxide and prostacyclin, which bind to the receptors of PASMCs and promote vasodilation and decreased cell proliferation. PASMCs in pulmonary hypertension have also been shown to also lose vascular tone balance through the inhibition of potassium ion channels, Kv1.5 and Kv2.1 [3], [4]. Endothelin-1 and platelet derived growth factor β (PDGF) receptor blockers are used to treat PAH but do not reduce death rates [5], [6]. MicroRNAs (miRs) are single-stranded RNA molecules, about twenty-two nucleotides, which regulates gene expression. They function through binding to the 3′ prime untranslated regions (3′UTR) of their target messenger RNA transcripts, thereby inhibiting protein synthesis. Alterations in the expression levels of various miRs in PAH have been reported [7], [8]. For example, miR-204 is down-regulated in PAH associated PASMCs [6]. Vascular smooth muscle cell proliferation is suppressed by increased expression of miR-145 and miR-143 through inhibition of Kruppel-like factor4 and Elk 1, respectively [9]. Treating PAH by correcting the levels of altered miR could be a potential therapeutic strategy. Both PAH and cancer are characterized by increased proliferation and resistance to apoptosis [2], [10]. miR-206 is down- regulated in many forms of cancers [11], [12]. Overexpression of miR-206 in HeLA cancer cells increases apoptosis by inhibiting Notch3 protein expression [13] and such over- expression plays a major role in PAH [14], [15]. *In vivo* experiments showed lower expression of miR-206 in hypoxia induced PAH mice when compared to control mice (Room air). Based on this evidence, the effects of miR-206 in modulating cell proliferation, apoptosis of PASMCs *in vitro,* and miR-206′s role in affecting PASMCs differentiation were investigated. We report for the first time that over expression of miR-206 decreases proliferation, increases apoptosis and induces smooth muscle cell differentiation markers (α-SMA and calponin) expression in human PASMCs. miR-206 also inhibits Notch3 protein expression levels and providing an explanation for increased Notch-3 levels in PAH.

## Materials and Methods

### Hypoxia Exposure

Approval of the study protocol was obtained from the Massachusetts General Hospital Institutional Animal Care and Use Committee with protocol number A3596-01. All mice were maintained in a specific-pathogen-free animal facility at Massachusetts General Hospital and animal experiments were carried out according to the provisions of the Animal Welfare Act, PHS Animal Welfare Policy, and the principles of the NIH Guide for the Care and Use of Laboratory Animals. Five age and sex-matched C57BL/6 mice were exposed to room air (21% FiO2) or hypoxia (10% FiO2) at sea level. All mice were 3 months old and weighed 22.5±2.0 grams. Mice were housed in a sealed chamber and the O2 concentration was maintained at 10% by controlling the inflow rate of compressed air and N2. The CO2 concentration was maintained at <0.4% with a CO2 absorbent. Gas samples were tested twice per day during the entire experimental period to monitor O2 and CO2 tension. The chamber was unsealed for less than 30 minutes twice per week to replenish food, replace CO2 absorbent and clean the cages.

### Measurement of Right Ventricular Systolic Pressure (RVSP)

Mice hemodynamic measurements were performed as previously described [16]. Mice were anesthetized by administration with ketamine and diazepam. A midline sternal skin incision was made from the second intercostal space to the xiphoid process and a 25-gauge needle was attached to a male/male luer slip connector. This was joined, in a successive order, a 18-gauge blunt needle, polyethylene 190 tubing (ID 1.19 mm; OD 1.70 mm), an 18-gauge blunt needle connected to a physiological transducer (Becton Dickinson DTX Plus DT-XX) via a two-way plastic stopcock. The needle was then inserted into the right ventricle by following a 45° trajectory between the right second and third intercostal space above the xiphoid process. Pressures were recorded on a Gould chart recorder (Model RS3400) with an embedded Gould transducer amplifier (Model 13-4615-50).

**Table 1:** Primers used in Real Time RT-PCR analysis.

Gene	Forward primer	Reverse primer
Calponin	GGCATCATTCTTTGCGAATTC	TGGTGATGGCCTTGATGAAG
α- Smooth muscle actin	CAACCGGGAGAAAATGACTC	GCGTCCAGAGGCATAGAGAG
Notch 3	AGGCCATGGTCTTCCCTTAC	ACACAGTCGCGGTTG
PCNA	AGGTGTTGGAGGCACTCAAG	CAGAGCCGGTTGTCAACTTC
GAPDH	CGGAGTCAACGGATTTGGTCGTAT	AGCCTTCTCCATGGTGGTGAAGAC

### Cell Culture

Human Pulmonary Artery Smooth Muscle Cells (hPASMCs) were obtained commercially (Cascade Biologics, Portland. OR) and cultured with Medium 231 with Smooth Muscle Growth Supplement (Life technologies, Grand Island, NY) and incubated at 37°C in a humidified 5% CO_2_ incubator. Cells at passage 3 or 4 were used for all the experiments.

### Isolation of Mouse PASMCs

Mouse PASMCs were derived from pulmonary arterioles using an iron oxide magnetic separation method as previously described [8], [17]. Ten mice were used for each group to isolate PASMCs.

### miR-206 Expression Level in Mouse PASMCs

Total RNA enriched with small RNAs, including miRNAs, was extracted from mouse PASMC using the mirVana™ miRNA Isolation Kit (Ambion, Foster City, CA). The extracted RNA was subjected to reverse transcriptase PCR using the TaqMan® MicroRNA Reverse Transcription Kit (Applied Biosystems, Carlsbad, CA). miR-206 amplification using TaqMan® MicroRNA Assays specific levels were measured by Real-Time PCR for miR-206 (Applied Biosystems, Carlsbad, CA), according to the manufacturer’s instructions. miRNA expression was normalized to the RNA U6B small nuclear (RNU6B) (Applied Biosystems, Carlsbad, CA).

**Figure 1:**
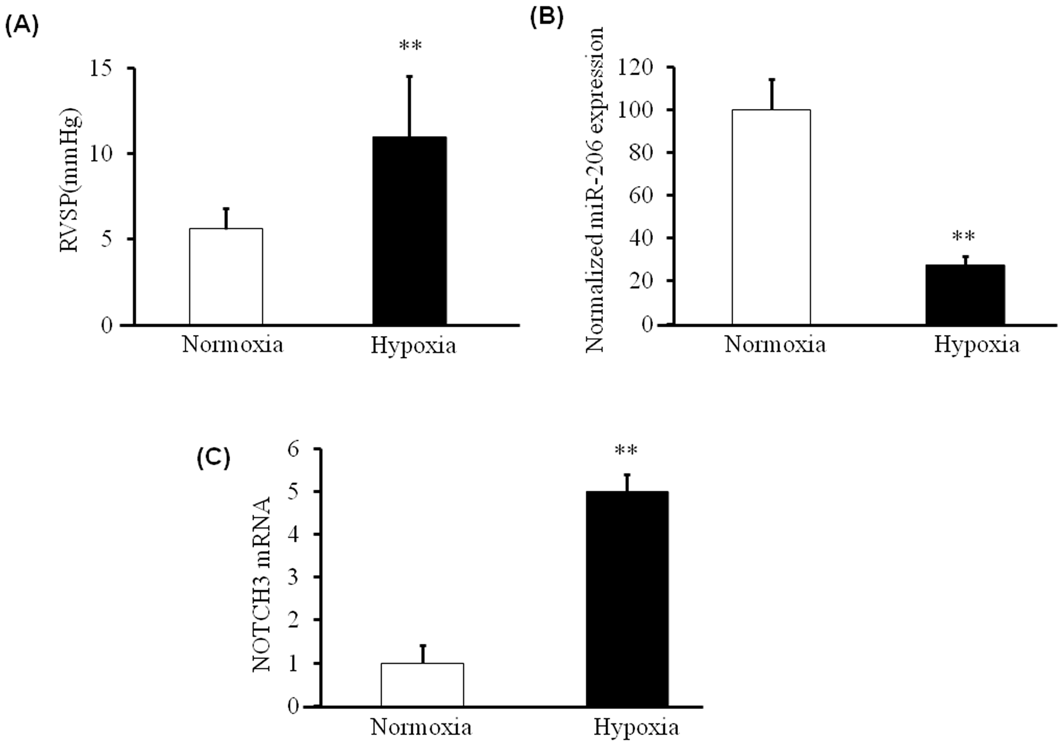
miR-206 is Down regulated in Hypoxia induced PAH mice. Mice were exposed to hypoxia or kept at normal room air. A) Right ventricle systolic pressure was significantly increased in mice exposed to hypoxic air compared to controls (n = 5) B) miR-206 levels were estimated in PASMCs isolated from mouse (n = 20) pulmonary arteries. A significant down regulation in the expression of miR-206 in hypoxic PAH mice compared to controls was observed. Values are represented as percentage of control C) Notch-3 mRNA levels were down regulated in PASMCs isolated from mice exposed to hypoxia compared to controls. P<0.05 was considered significant ** = p<0.01.

### Transfection of hPASMCs

miR-206 plasmid was purchased from Addgene (Cambridge, MA.). Anti-miR 206 and negative control miRNA were obtained from Dharmacon (Thermo Fischer Scientific, Lafayette, CO). hPASMCs and mouse PASMCs were transfected with 500 ng of miR-206 or 10 pm of antimiR-206 or negative control miR using a Primary P1 Nucleofection Kit and 4D Nucleofector machine (Lonza, GA).

### Proliferation Assay

Transfected hPASMCs were seeded (10^4^) in 96 well plates supplemented with Medium 231 and incubated for 48 h. Proliferation assay was performed using CellTiter 96 Cell Proliferation Assay kit (Promega, Madison, WI) as per the manufacturer instructions. After 48 h of incubation, 20 µl of 3-(4,5-dimethylthiazol-2-yl)-5-(3-carboxymethoxyphenyl)-2-(4-sulfophenyl)-2H-tetrazolium,innersalt (MTS)/phenazine methosulfate (PMS) mixture was added to cells in each well of the 96 well plate and incubated for 4 h at 37°C in 5%CO_2_. The absorbance of each plate was read at 490 nm using a plate reader.

### Wound Healing Assay

Transfected hPASMCs (2×10^5^) were seeded in12-well plates and incubated at 37°C in 5%CO_2_ until a complete monolayer was formed. Cells were co-transfected with a GFP vector to track the migration of cells. A 1 mL pipet tip was used to scratch through the middle of the monolayer, creating an artificial wound field. Photographs were taken, via fluorescence microscopy, at 0 and 24 hours to assess the level of migration in each group of transfected cells. Migration was quantified by counting the total number of cells that migrated toward the original wound field.

**Figure 2:**
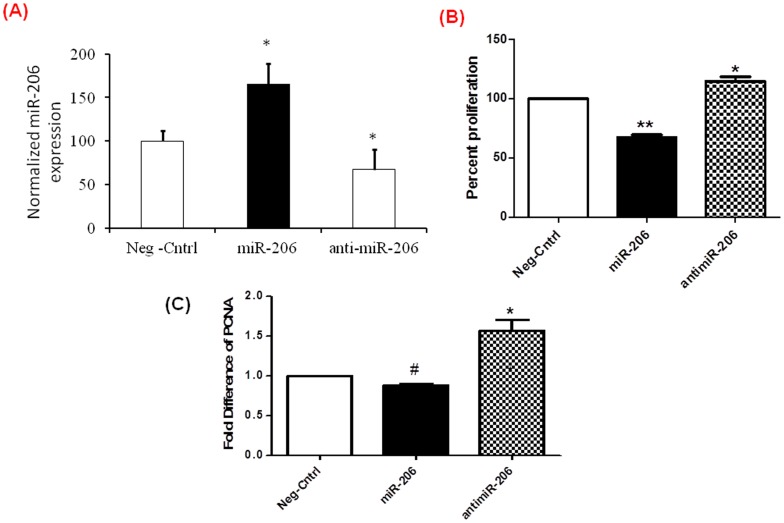
miR-206 down regulates proliferation of human PASMCs. hPASMCs were transfected with miR-206, antimiR-206 or control miRs and 48 hrs post transfection A) Analyzed for miR-206 levels by real time analysis B) MTT assay showing decreased proliferation of hPASMCs treated with miR-206 and increased proliferation of hPASMCs treated with antimiR-206 compared to controls. Results represented are the average of three independent experiments normalized as percent control ±SEM. C) Real time PCR analysis for proliferation marker PCNA indicates decrease expression of proliferation markers in cells transfected with miR-206 and increased expression of proliferation markers in cells transfected with antimiR-206. Results presented are show significant difference respective to controls after normalizing with GAPDH. All experiments were repeated at least 3 independent times, a paired two– tailed t-test was done and p≤0.05 was considered statistically significant **p<0.01, *p<0.05, #p = 0.504. p≤0.05 is considered statistically significant.

### Migration Assay

Migration assay was performed using a 96 well cell migration assay (Cultrex, Trevigen, Inc, Gaithersburg, MD). hPASMCs were transfected with control or miR-206 or anti-miR 206 and migration assay was performed as per the manufacturer instructions.

### Apoptosis Assay

Apoptosis of hPASMCs transfected with the mir206 construct, antimiR-206 or the negative control was assessed by TUNNEL assay. Transfected hPASMCs were seeded (2×10^4^) in 8-well chamber/slides (Millipore, Billerica, MA) with supplemented Medium231. After 24 hours, TUNNEL assay was performed using the ApopTag Flourescin Kit (Millipore, Billerica, MA) as per the manufacturer instructions. The number of TUNNEL positive cells in 2–4 fields for each treatment group were counted using the Olympus fluorescent microscope and expressed as percent apoptotic cells.

### Caspase 3 Activity Assay

Caspase 3 activity assay was performed using Caspase-Glo 3/7 Assay kit (Promega, Madison, WI). hPASMCs were transfected with negative control, miR-206 or anti miR-206 over expressing plasmids and Caspase 3 activity was assessed as per the manufacturer instructions.

**Figure 3:**
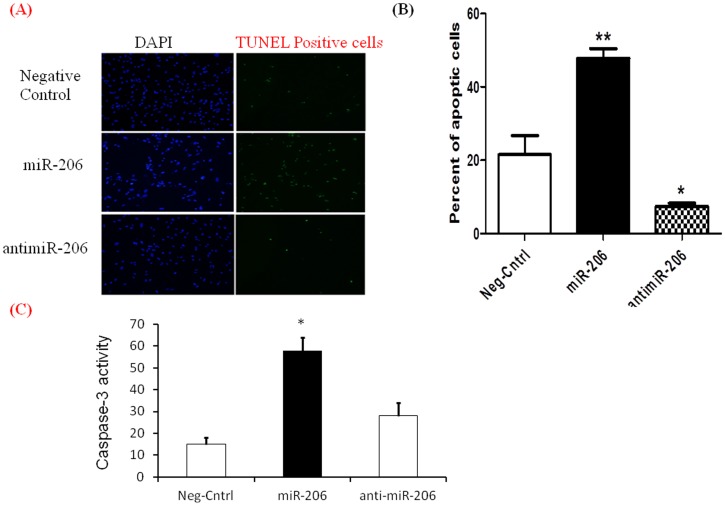
miR-206 increases apoptosis in hPASMCs. Apoptosis of hPASMCs transfected with miR-206, anti miR-206 or the negative control was assessed by TUNNEL assay and Caspase 3 activity assay. A) Fluorescent micrographs indicating TUNNEL positive cells (green) and nuclei counterstained with DAPI (blue) B) Quantitative analysis of TUNEL apoptotic cells expressed as percent apoptotic cells compared to controls. C) Caspase 3 activity in hPASMCs transfected with miR-206, anti miR-206 or negative control. All experiments were repeated at least 3 independent times, a paired two tailed t-test was done and p≤0.05 was considered statistically significant **p<0.01, *p<0.05.

### Collagen Contraction Assay

Collagen contraction assay was performed using cell contraction assay kit (BioLabs, Sandiego, CA). PASMCs isolated from mice exposed to hypoxia were transfected with control vector or miR-206 or anti-miR 206 and were re suspended in collagen matrix gel. Contraction assay was performed as per the manufacturer instructions.

### Real Time RT-PCR Analysis

The relative expression levels of Proliferating Cell Nuclear Antigen (PCNA), calponin, α-Smooth Muscle Actin (SMA) and NOTCH-3 was determined by real time PCR analysis using primers. Total mRNA was isolated using Trizol according to the manufacturer’s protocol (Life technologies, Grand Island NY). 1 µg of RNA was converted into cDNA using the I Script cDNA synthesis kit followed by real time PCR using the Syber Green master mix (Biorad, Hercules, CA). All primers (Table 1) were purchased from Integrated DNA Technologies Inc (Coralville, IA) and reconstituted in nuclease free distilled water. The final concentrations of the primers used in real time assays were 1 µM. All experiments were repeated three independent times in duplicates. Relative fold differences in mRNA levels were compared with the normalized controls using the delta delta Ct method.

**Figure 4:**
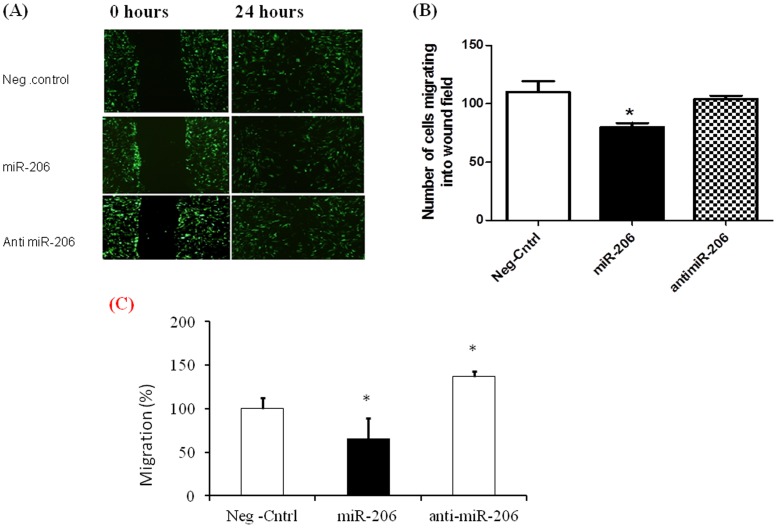
miR-206 decreases migration of hPASMCs. Migration of hPASMCs transfected with miR-206, antimiR-206, and control miR was assessed by wound healing and migration assay. hPASMCs were co-transfected with GFP to aid in better visualization of cells. A) Representative micrographs of wound healing assays at 0 hrs and 24 hrs after creating a wound field. B) Quantitative assessment of number of cells migrating into the wound field indicating that miR-206 significantly reduces migration of hPASMCs compared to controls. C) Migration of hPASMCs transfected with miR-206, antimiR-206, and control miR represented as % migration. Data presented represent average±SEM. All experiments were repeated at least 3 independent times, a paired two tailed t-test was done and p≤0.05 was considered statistically significant.

### SDS PAGE and Western Blot

Transfected hPASMCs were cultured for 48 h, washed with PBS and lysed in RIPA buffer (Thermo scientific, Rockford, IL). The amount of protein in the lysates was estimated using the bicinchoninic acid (BCA) assay kit (Pierce, Rockford, IL). Lysates with equal protein concentrations were mixed with 4× Laemmli Buffer (Boston BioProducts, Ashland MA), heated at 95°C for five minutes and subjected to electrophoresis in a 10% polyacrylamide gel. Proteins were then transferred onto a nitrocellulose membrane (Millipore, Billerica, MA) and blocked with 5% dry nonfat milk in 1× TBS Tween (10 Mm Tris-HCl, pH 7.6, 150 mM NaCl, 0.05% Tween-20) for 1 h. Each membrane was probed with a specific primary antibody overnight in 5% milk (in 1× TBST). SMC markers like calponin and α-smooth muscle actin (Abcam, Cambridge, MA) were used at a dilution of 1∶2000. Notch-3 (Cell signaling, Danvers, MA) was used at a dilution of 1∶2000 as recommended by the manufacturer instructions. After washing, 3 times in TBST, the secondary antibody, rabbit anti-horseradish peroxidase (Jackson ImmunoResearch Laboratories, West Grove, PA), was added (1∶1000) and incubated for 1 hour at room temperature. After 3 washings with TBST, membranes were developed using chemi luminescence kit (Thermo scientific, Rockford, IL).

**Figure 5:**
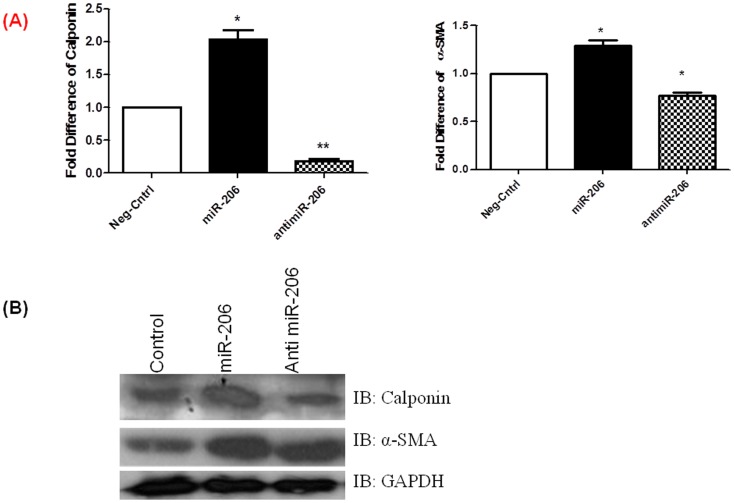
miR-206 increases expression of SMC markers. Expression of SMC differentiation markers α-smooth muscle actin (SMA) and calponin was analyzed in hPASMCs transfected with miR-206, antimiR-206 and controls by A) Real time PCR and B) Western blotting. MiR-206 transfected cells show increased expression of SMC markers α-SMA and calponin compared to controls, while antimiR-206 transfected cells show reduced expression of SMC markers. Real time data are presented as the average fold difference compared to controls after normalizing with GAPDH. All experiments were repeated at least 3 independent times, a paired two tailed t-test was done and p≤0.05 was considered statistically significant. **p<0.01, *p<0.05. Densitometric analysis of the immunoblot is included in the supplementary data.

### Statistical Analysis

All experiments were repeated three independent times. All statistical analyses were done and graphs were plotted using the GraphPad Prism 5 software. A two-tailed student t-test was used and p<0.05 was considered statistically significant.

## Results

### miR-206 Levels are Down Regulated in Hypoxia Induced PAH Mice

Pulmonary hypertension was induced in mice by exposing them to 10% hypoxia for six weeks. Mean right ventricular systolic pressure was significantly (p<0.05) elevated in mice exposed to hypoxic versus normal air (Figure 1A). PASMCs were isolated to quantitate miR levels from hypoxic PAH and control mice. A significantly (p<0.05) decreased expression levels of miR-206 was observed in PASMCs of hypoxia induced PAH mice compared to controls (Figure 1B). Notch3 mRNA levels were up-regulated in PASMCs of PAH mice (Figure 1C).

**Figure 6:**
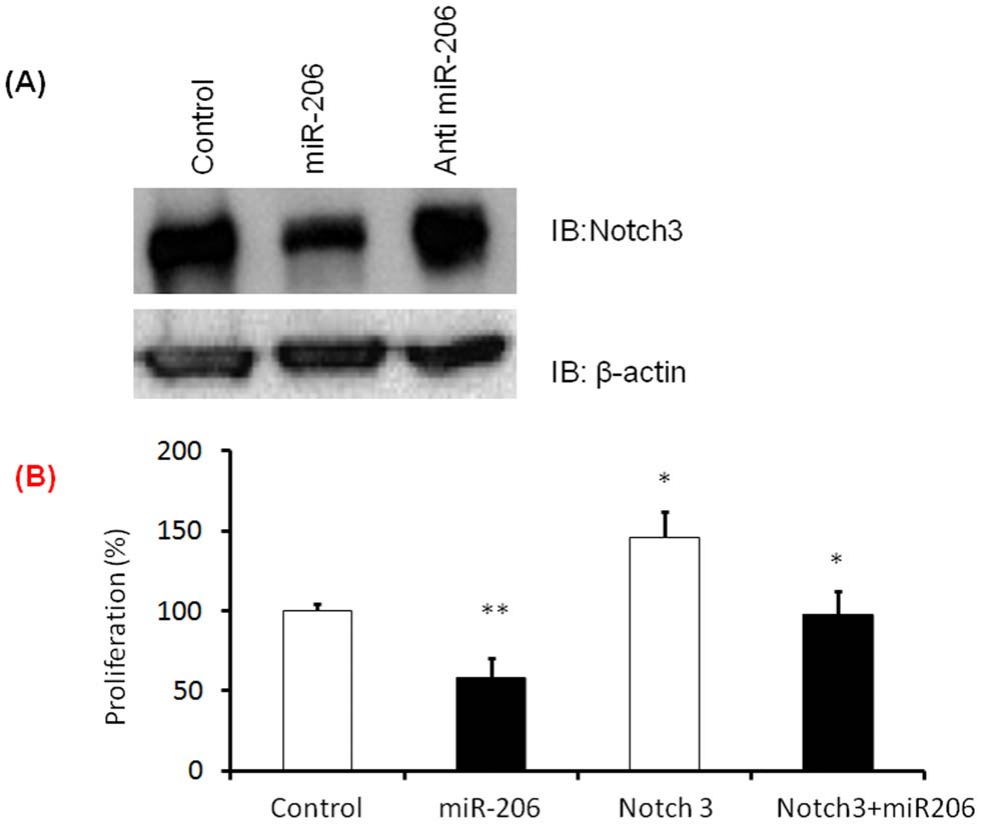
miR-206 reduces Notch-3 expression and rescues the proliferative phenotype by inhibiton of Notch 3 in hPASMCs. Notch-3 expression levels were assessed in hPASMCs transfected with miR-206, antimiR-206 and control miR A) A representative western blot is shown wherein miR-206 transfected cells show decreased expression of notch-3 compared to controls while antimi-206 treated samples show a higher expression of Notch3 compared to controls. Densitometric analysis of the immunoblot is included in the supplementary data. B) Rescue experiments were performed using hPASMCs transfected with miR-206 and Notch 3 over expressing plasmids or both and proliferation was assessed using 96 cell proliferation assay kit. All experiments were repeated at least 3 independent times, a paired two tailed t-test was done and p≤0.05 was considered statistically significant. **p<0.01, *p<0.05.

### miR-206 Over Expression Decreases Proliferation in hPASMCs

miR-206 expression levels were found to be significantly lower in mice with pulmonary hypertension induced by hypoxia as compared to normoxic mice and suggest that miR-206 may play a role in regulating the proliferative phenotype of the pulmonary vasculature. To determine the functional role of miR-206, hPASMCs were transfected with a miR negative control, miR-206 plasmid vector, or antimiR-206 and the levels of miR-206 was analyzed using q PCR (Figure 2A).Overexpression of miR-206 significantly decreased smooth muscle cell proliferation by 37%, while inhibition of miR-206 by antimiR-206 significantly increased proliferation by 15% compared to controls (Figure 2B). Real time PCR analysis of proliferation marker PCNA also showed a similar decrease when compared to controls (Figure 2C). These results support the hypothesis that miR-206 expression decreases proliferation in hPASMCs.

**Figure 7:**
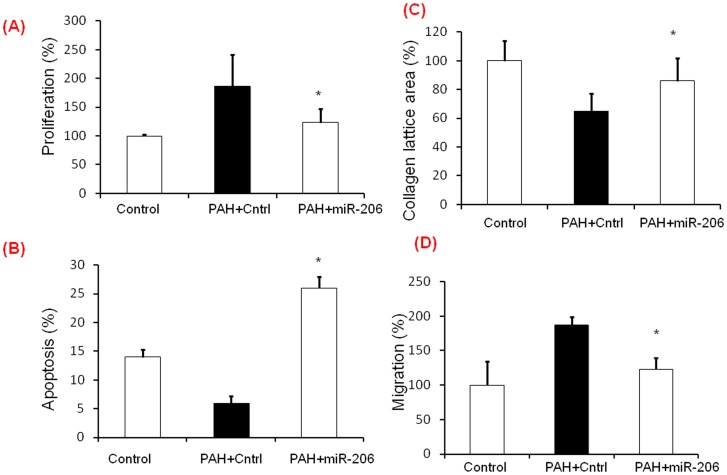
miR-206 decreases proliferation, migration, contraction and increases apoptosis in PASMC’s of hypoxia induced PAH mice. PASMCs were isolated from mice exposed to hypoxia and functional effects of miR-206 on proliferation, apoptosis, contraction and migration were analyzed post transfection. A) Proliferation assay showing significant decrease in proliferation in miR-206 overexpressed mice PASMCs B) Increased % apoptotic cells in miR-206 transfected mice PASMCs compared to control transfected cells C) Contraction assay showing significant increase in collagen lattice area in miR-206 transfected mice PASMCs compared to control transfected cells D) Migration assay showing a significant decrease of % migrated cells in miR-206 overexpressed mouse hypoxic PASMCs. All experiments were repeated at least 3 independent times, a paired two tailed t-test was done and p≤0.05 was considered statistically significant. *p<0.05.

### miR-206 Overexpression Increases Apoptosis in hPASMCs

One of the important features of PAH is decreased apoptotic activity in hPASMCs. We investigated the effects of miR-206 over expression on apoptosis in hPASMCs by TUNEL staining.miR-206 treated samples showed increased numbers of TUNEL positive cells 24 hours after transfection, indicating increased apoptotic activity (Figure 3A). There was a 2.5-fold increased percentage of apoptotic cells in hPASMCs over-expressing miR-206. The number of apoptotic cells was also significantly reduced in hPASMCs transfected with antimiR-206 (Figure 3B). We further assessed apoptosis using Caspase 3 assay kit as per the manufacturer instructions. Our data shows a significant increase in Casapase 3 activity in miR-206 over expressing hPASMCs compared to controls. hPASMCs transfected with anti miR-206 showed decreased apoptosis (Figure 3C). These results indicate that over-expression of miR-206 can increase apoptotic activity in hPASMCs and its down-regulation reduced apoptosis of hPASMCs.

### miR-206 Over Expression Decreases Cellular Migration in hPASMCs

Vascular remodeling and increased cellular migration is another histopathology finding displayed in PAH (16). The wound healing (migration) assay was performed in hPASMCs transfected with the respective miRs. After creating a wound field, photographs were taken at 0 and 24 hours via fluorescent microscopy to assess the degree of migration in transfected group. Cells over-expressing miR-206 were found to migrate towards the center of the wound at a lower rate as compared to the controls, which almost completely enclose the wound field after 24 hours (Figure 4A). Quantitatively, there was a significant decrease in the number of cells that migrated towards the interior of the initial wound field at 24 hours in miR-206 transfected hPASMCs (Figure 4B). There were no significant differences in the number of migrating cells between the anti miR-206 samples and the controls. Additionally, we analyzed the functional effects of miR-206 on migration using a kit as per the manufacturer instructions (Figure 4C). Our data showed a significant decrease in % migration of miR-206 transfected hPASMCs compared to controls. hPASMCs transfected with anti miR-206 showed a significant increase in migration rates compared to control. These results indicate that over-expression of miR-206 decreases cellular migration in hPASMCs.

### miR-206 Over Expression Induces hPASMCs Differentiation Markers

hPASMCs take on a de differentiated proliferative phenotype in PAH and are unable to differentiate or contract that is necessary for the normal functioning of the pulmonary vasculature. Real time PCR and immuno-blotting was done to assess the relative expression of smooth muscle differentiation markers, α smooth muscle actin and calponin. hPASMCs that over-expressed miR-206 showed higher α-SM actin and calponin mRNA expression (Figure 5A) and protein (Figure 5B and supplementary figure S1A&B) levels compared to controls, while those expressing antimiR-206 have decreased expression levels of these same proteins. These results indicate that miR-206 plays a role in differentiating hPASMCs.

### miR-206 Inhibits Expression of Notch3 in hPASMCs

miRs bind to the 3′ untranslated regions (3′UTR) of target messenger RNA and inhibit their translation. miR-206 targets the receptor protein, Notch3, found to play a role in tumorogenesis [13]. Notch3 is over-expressed in PAH vascular smooth muscle cells [14] but the cause of this steady state increase is unknown. Since miR-206 is known to target Notch3 expression in breast cancer, Notch3 expression levels in miR-206 transfected cells were assessed via immuno-blotting. hPASMCs that over expressed miR-206 were found to have lower Notch3 protein levels as compared to controls, while those transfected with antimiR-206 had increased expression levels of this protein (Figure 6A and supplementary figure S2). These results indicate that miR-206 inhibits Notch3 expression in hPASMCs. Further to assess miR-206 mediated regulation of Notch 3, we performed rescue experiments where in, hPASMCs were transfected with either miR-206 or Notch 3 or both and post transfection, we performed proliferation assay. Our data shows a significant decrease in proliferation in miR-206 transfected hPASMCs when compared to control transfected cells. Over expression of Notch 3 alone in hPASMCs increased proliferation as expected when compared to control transfected hPASMCs. However, co transfection of Notch 3 and miR-206 in hPASMCs resulted in a significant decrease in proliferation when compared to Notch3 transfected cells (Figure 6B). This data suggests that, miR-206 rescues Notch3 mediated proliferative effects in hPASMCs.

### miR-206 Over Expression Decreased Proliferation, Migration, Contraction and Increased Apoptosis in PASMC’s of Hypoxia Induced PAH Mice

Pulmonary hypertension was induced in mice by exposing them to 10% hypoxia for six weeks. PASMCs were isolated to assess the functional effects of over expression of miR-206 on proliferation, migration, contraction and apoptosis. PASMCs isolated from mice exposed to hypoxia and room air were cultured in medium 231 as per the manufacturer instructions. Cells were transfected with miR-206 containing plasmid (PAH+miR-206) or control vector only (PAH+Cntrl) and functional assays were performed as described in the methods section. PASMCs isolated from mice exposed to room air served as controls (Control). Proliferation was significantly suppressed in miR-206 over expressed PASMC’s isolated from mice exposed to hypoxia when compared to control transfected mice PASMCs (Figure 7A). Apoptosis was significantly increased in miR-206 over expressed PASMCs isolated from hypoxia exposed mice (Figure 7B) compared to control transfected PASMCs (PAH+miR-206). Percentage collagen lattice area was assessed in miR-206 overexpressed PASMCs isolated from hypoxia exposed mice. PASMCs with miR-206 showed less contractibility when compared to control transfected PASMCs (Figure 7C). Migration was significantly decreased in PASMCs over expressing miR-206 compared to control vector transfected cells (Figure 7D). These results suggest that miR-206 over expression decreased proliferation, migration, contraction and increased apoptosis in PASMC’s of hypoxia induced PAH mice.

## Discussion

Various in-vivo and in-vitro studies show that enhanced cell proliferation and apoptosis resistance state of PASMCs is a key feature in the progression of PAH [1], [2], [9]. Mutations in the bone morphogenetic protein receptor-II are associated with familial PAH [18]. However, lack of association between BMPR-II mutations in non-familial PAH cases [19] suggest that other signaling events may also be involved in the pathogenesis of PAH. miRs appeared to be important in PAH by various reports published recently [9], [20], [21]. miR-206 has been studied in various cancer cells [12], [13], [22] **,** however, miR-206 has not been studied in PAH hPASMCs. miR-206 is significantly down-regulated in hypoxic PAH mice compared to controls as illustrated in this study. Our *in-vitro* studies using hPASMCs show that over-expressing miR-206 decreases proliferation and increases apoptosis while reducing levels of miR-206 has opposing effects, indicating that reduced expression levels of miR-206 may be contributing to the observed hyper-proliferative state of hPASMCs in PAH. miR-206 is increases expression of SMC differentiation markers, SMA and calponin and suggest that it may also play a crucial role in regulating SMC differentiation.

Notch3 signaling is important in the development of PAH [14]. Interactions between the Notch3 target gene and downstream BMPR target genes are reported [23], [24], further implicating the importance of the Notch3 signaling and the crosstalk between Notch3 and BMPR-II signaling in vascular pathobiology. Notch3 is expressed only in the vascular smooth muscle cells of arteries but not veins [15], [25], [26] and is thought to be important in maintaining SMC proliferation and a dedifferentiated state of the cells [27], [28]. Notch3 levels are upregulated in lungs from human PAH [14] but the cause of this dysregulation and higher steady state expression is not understood. As demonstrated in the study, expression of miR-206 down regulates Notch3 levels in hPASMCs, and when miR-206 is abrogated by using anti miR-206, Notch3 expression increases compared to controls. Therefore, a biological explanation for an increase in Notch3 levels in PASMCs in PAH by down regulating miR-206 is provided for the first time. A similar role for miR in regulating key molecule in PAH is reported [7]. miR-17 is highly expressed in PAH and directly down regulates BMPR-II levels, providing evidence for loss of BMPR-II function in non-familial PAH [29].

PAH and cancer share similarities for both diseases are characterized by increased proliferation and resistance to apoptosis. miR-206 increases apoptosis and inhibits tumor formation [13] in cancer cells by directly targeting Notch3. Notch3 over-expression decreases apoptosis in vascular smooth muscles through downstream activation of c-FLIP, which inhibits the fas-ligand apoptotic signaling pathway. This ultimately could lead to increasing cell numbers [30]. Notch3 is also believed to be important in maintaining a proliferative state of PASMCs [27], [31]. The findings as demonstrated in this study, over-expression of miR-206 reduces proliferation and increases apoptosis of hPASMCs are consistent with these known functions of Notch3. miRs are known to regulate many genes, and it is possible that effect of miR206 on increased proliferation and reduced apoptosis could be because of its effect on other proliferation associated genes such as VEGF or cyclin D2, as reported earlier [32].

miR-206 induces differentiation of myoblasts [33]. Therefore, relative expression levels of hPASMCs phenotypic markers were assessed to measure the effect of miR-206 on hPASMCs differentiation. A significant increase in the expression of smooth muscle phenotypic markers, α -SMA and calponin, was observed in cells over-expressing miR-206 also induces differential expression. This suggests that miR-206 may be an important regulator in the differentiation of hPASMCs. The expression of the constitutively active form of Notch3 intra-cellular domain down regulates smooth muscle cell-specific α-actin, myosin, calponin, and smoothelin in human aortic smooth muscle cells [34]. The effects of mir-206 on the SMC phenotype could be attributed, at least in part, to its effect on Notch3, a known factor that represses SMC differentiation and involved in the pathogenesis of PAH development [28], [34]. These results suggest that miR-206 is a potent regulator of PASMCs and a disturbance in its expression levels can lead to PAH.

In conclusion, miR-206 appears to be a key regulator of the PASMCs function in the pulmonary vasculature. Suppressed levels of miR-206 observed in PAH mice might facilitate the proliferation and migration of PASMCs and enhance their resistance to apoptosis, leading to the thickening of the medial layer of pulmonary artery. These reduced miR-206 levels could also contribute to the observed increased Notch3 levels in PAH. Notch3 inhibitors are toxic in nature [35], [36] and nontoxic molecules that modulate Notch are in demand. Regulation of Notch3 by miR-206 suggests that miR-206 could be used as a potential therapeutic molecule to modulate Notch3 functions, which promotes PAH development.

## Supporting Information

Figure S1 **A) Densitometric analysis of immunoblot probed for calponin represented in** **Figure 5B** **.** B) Densitometric analysis of immunoblot probed for α-SMA represented in Figure 5B.
(TIF)

Figure S2 **Densitometric analysis of immunoblot probed for Notch 3 represented in** **Figure 6A** **.**
(TIF)
