# Supplementary figures and images for: Mir-206 Regulates Pulmonary Artery Smooth Muscle Cell Proliferation and Differentiation
Source: PLoS One. 2012 Oct 10;7(10):e46808. doi: 10.1371/journal.pone.0046808 (PMC3468623; doi:10.1371/journal.pone.0046808)

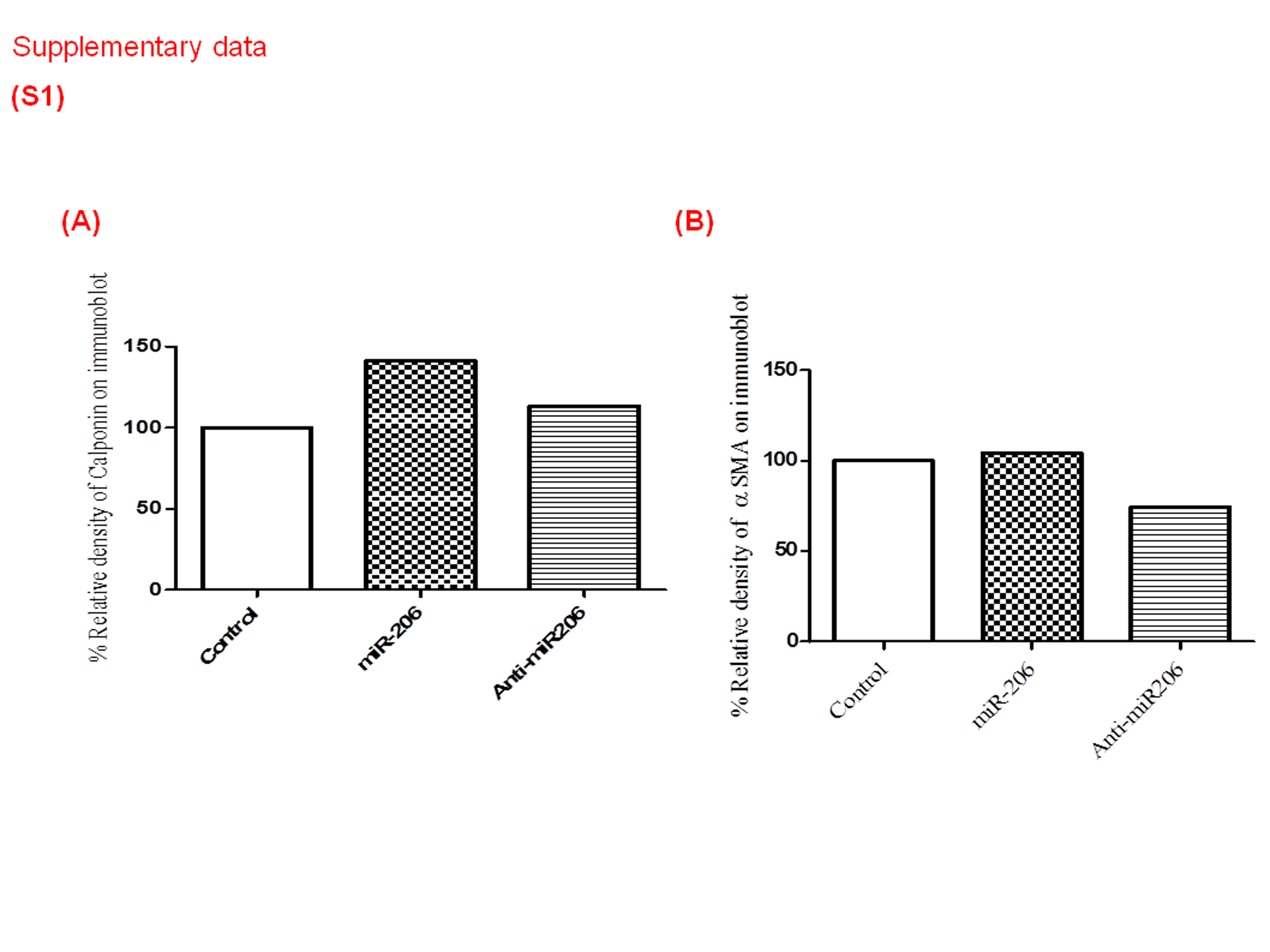

Supplement: Figure S1 — A) Densitometric analysis of immunoblot probed for calponin represented in Figure 5B . B) Densitometric analysis of immunoblot probed for α-SMA represented in Figure 5B. (TIF) [file pone.0046808.s001.tif]

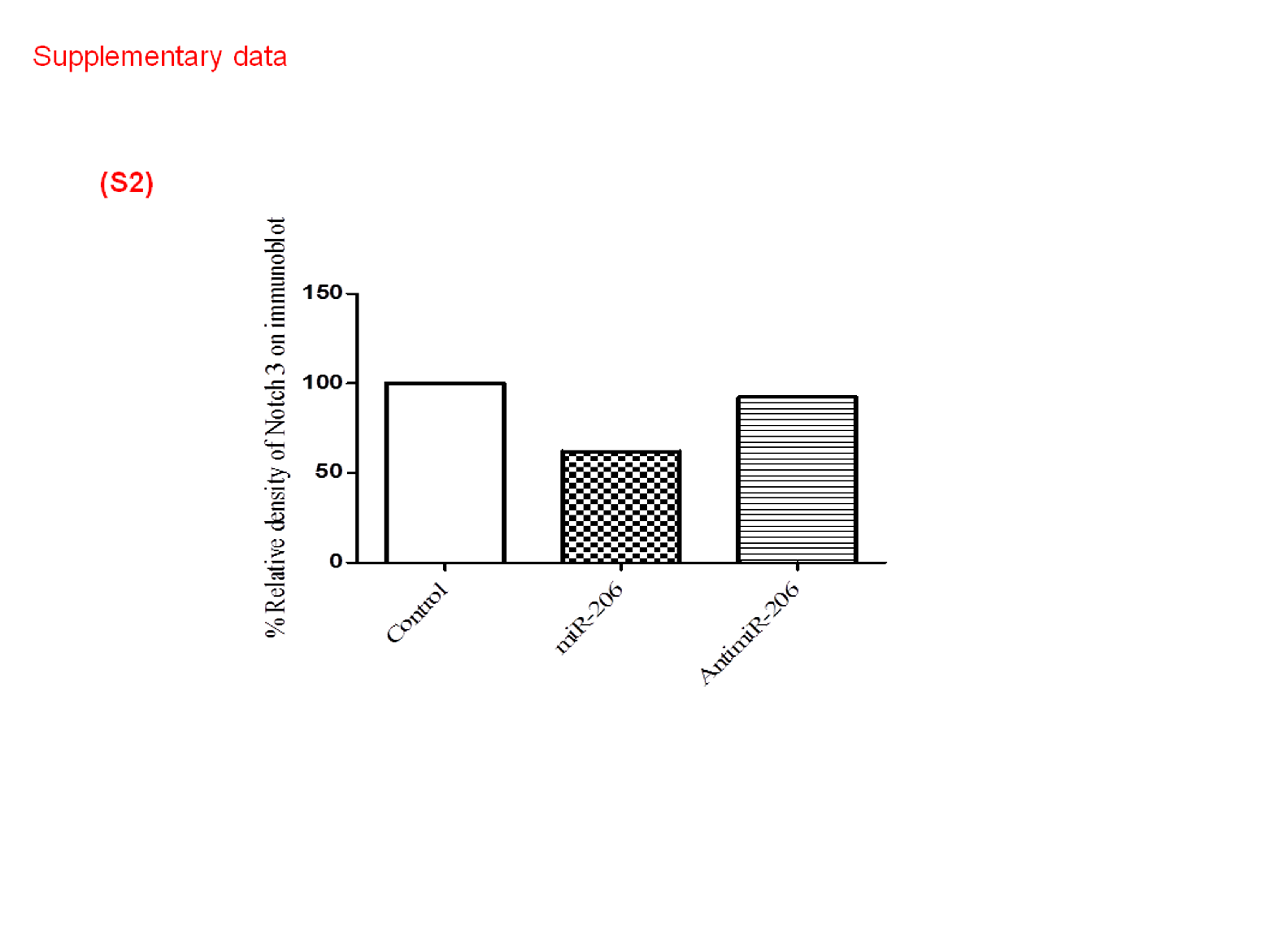

Supplement: Figure S2 — Densitometric analysis of immunoblot probed for Notch 3 represented in Figure 6A . (TIF) [file pone.0046808.s002.tif]
